# Real-Time Monitoring of Personal Protective Equipment Adherence Using On-Device Artificial Intelligence Models

**DOI:** 10.3390/s25072003

**Published:** 2025-03-22

**Authors:** Yam Horesh, Renana Oz Rokach, Yotam Kolben, Dean Nachman

**Affiliations:** 1 Faculty of Medicine, Hebrew University of Jerusalem, Jerusalem 9112001, Israel; renana.oz@mail.huji.ac.il (R.O.R.); yotam@4kosh.com (Y.K.); 2 Heart Institute, Hadassah Medical Center, Jerusalem 9112001, Israel

**Keywords:** computer vision, personal protective equipment, adherence monitoring, single-board computer, edge computing

## Abstract

Personal protective equipment (PPE) is crucial for infection prevention and is effective only when worn correctly and consistently. Health organizations often use education or inspections to mitigate non-compliance, but these are costly and have limited success. This study developed a novel on-device, AI-based computer vision system to monitor healthcare worker PPE adherence in real time. Using a custom-built image dataset of 7142 images of 11 participants wearing various combinations of PPE (mask, gloves, gown), we trained a series of binary classifiers for each PPE item. By utilizing a lightweight MobileNetV3 model, we optimized the system for edge computing on a Raspberry Pi 5 single-board computer, enabling rapid image processing without the need for external servers. Our models achieved high accuracy in identifying individual PPE items (93–97%), with an overall accuracy of 85.58 ± 0.82% when all items were correctly classified. Real-time evaluation with 11 unseen medical staff in a cardiac intensive care unit demonstrated the practical viability of our system, maintaining a high per-item accuracy of 87–89%. This study highlights the potential for AI-driven solutions to significantly improve PPE compliance in healthcare settings, offering a cost-effective, efficient, and reliable tool for enhancing patient safety and mitigating infection risks.

## 1. Introduction

Medical staff are the main vector for spreading contagious infections within hospitals, playing a role in initiating or amplifying nosocomial outbreaks [1]. Lack of adherence to standard precautions regulations remains one of the most influential factors for transmission of hospital-acquired infections, requiring rigorous training programs to ensure compliance [2]. In departments housing complex patients for extended periods, inoculation with highly resistant pathogens could extend stays by 15.8 days [3]. Thoroughly enforcing infection control measures has significantly reduced contamination and contraction among both the healthcare staff [4] and the patients themselves. However, the methods currently used to ensure such adherence are inefficient, and their deployment is limited by the availability of human resources [4]. In healthcare settings, the significance of PPE cannot be overstated. It forms the cornerstone of infection control, a factor that not only influences patient outcomes but also operational costs. Traditional methods for monitoring PPE adherence are often manual, sporadic, and prone to human error, making them insufficient for ensuring consistent compliance.

Over the last few years, a new opportunity to reduce the burden of the repetitive task of monitoring adherence has emerged in the form of artificial intelligence and computer vision [5]. The COVID-19 pandemic even further accelerated the worldwide utilization of different methods to ensure such adherence. Unfortunately, these solutions were mostly centered around the “mask-wearing” aspect of the pandemic, implementing cutting-edge verification technologies to handle a wide range of common daily [6] and urban [7] situations with 98.2% and 96% accuracy, respectively. While performing well, they were not suited for the broader adherence validation required in the healthcare environment. Furthermore, these AI solutions rely on a constant network connection, using a remote processing unit to compute the automated predictions

We propose a custom-tailored solution, allowing us to automatically detect PPE adherence in a wider context. Unlike contemporary solutions that concentrate on individual components like mask wearing, our approach offers a holistic monitoring framework. It encompasses the entire set of protective gear, including masks, gloves, and gowns, in accordance with established infectious disease prevention protocols [8]. Our approach is optimized to run on an offline single-board computer (SBC), as studies indicate promising avenues for future energetic improvements [9]. This work’s contribution lies in the implementation of a computer vision system that ensures the real-time, automated, and comprehensive monitoring of adherence. By employing a single-board computer for inference, the system not only delivers instant feedback but also upholds stringent data privacy standards. This reduces infrastructure costs and shortens latency times while retaining maximal user privacy and improving safeguards against external cyber threats [10].

## 2. Materials and Methods

A Raspberry Pi (Cambridge, UK) SBC was used for the physical inference setup [11,12]. To build and train our AI model, we used TensorFlow (version 2.14.1) [13] (Google). To facilitate the training, we implemented transfer learning [14] with a MobileNetV3 [15] (small-075-224 feature vector, version 5) extraction model, which offered the fastest frames-per-second throughput.

LiteRT (formerly TensorFlow lite, version 2.14.0), a TensorFlow submodule designed to create efficient models for resource-constrained hardware, was used to optimize the model.

### 2.1. System Design

#### 2.1.1. Model Pipeline

**Figure 1:**
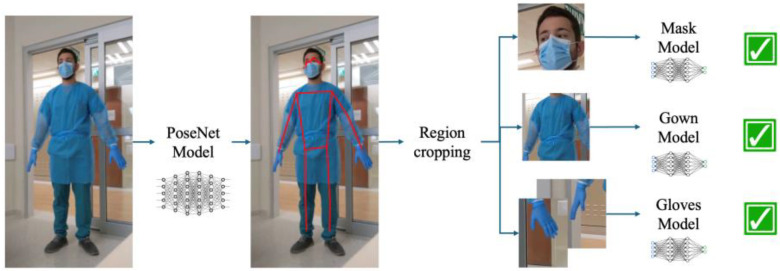
Illustration of the processing pipeline from image to classification results.

We designed a custom model processing pipeline that involves 3 steps (Figure 1):

1. Run the image through a pose estimation model to detect anatomical landmarks (eyes, nose, ears, shoulders, arms, hips, knees, and ankles).
2. Use these landmarks to segment and crop sections containing the relevant regions of interest for the protective equipment: palms, torso, and face.
3. Feed the cropped images into custom binary neural networks that can infer whether the protective gear is worn or not, being a mask, gloves, or a gown.

#### 2.1.2. Region of Interest Extraction

**Figure 2:**
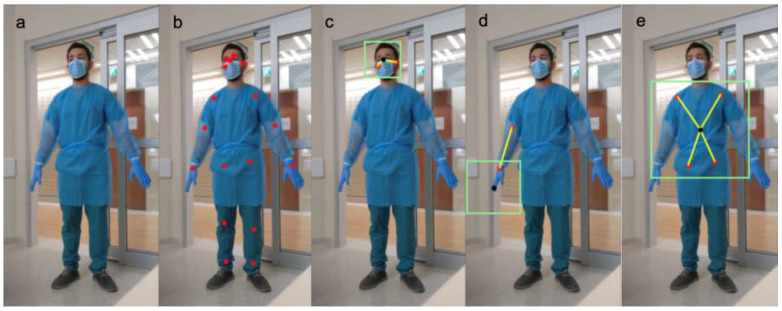
(**a**) Full body image, (**b**) MoveNet estimated landmarks, (**c**) cropped face region, (**d**) cropped palm region, (**e**) cropped torso region. Legend: landmarks (red dots); distance scale (yellow lines); region center (black dot); region border (green rectangle).

We segmented the pose (Figure 2a) with the pretrained TensorFlow Lite MoveNet [16] model, selecting the most efficient version of the model, even at the cost of accuracy. Its purpose is only to estimate landmark locations (Figure 2b), since the next steps add sufficient overlapping coverage. Instead of asking the model to detect PPE in the whole image, we simplify the task by training the custom models to detect a single item in each extracted regional image. This approach achieves accurate results with less training data [17].

Cropped sub-images were obtained using geometric algorithms, with spatially relevant landmarks and coefficients derived through trial and error:

- Face region (landmarks for right ear, left ear, and nose) (Figure 2c)
  The average Euclidean distance between each ear and the nose served as a measurement scale to crop a square around the nose, with a factor of 1.2 times the distance scale.
- Palm region (landmarks for right and left, elbow and wrist) (Figure 2d)
  The Euclidean distance between the elbow and wrist was the measurement scale. The palm center was calculated as a linear interpolation along a line connecting the elbow through the wrist, continuing 0.5 times the measurement scale. Then, a square crop is made around that pinpoint, with dimensions equal to the 1.6 times the measurement scale.
- Torso region (right and left shoulder and hip landmarks) (Figure 2e)
  The Euclidean center of mass was calculated using all 4 landmarks, from which a square was cropped with dimensions equal to 0.83 times the distance to the furthest landmark.

#### 2.1.3. AI Model Architecture

Three models were trained for custom equipment detection, one for each region, sharing the same structure. A pretrained MobileNetV3 [16] feature extraction model converted 224 × 224 color images into a one-dimensional vector of size 712. A dropout layer with a rate of 0.2, followed by a 712 linear matrix layer, a ReLU (rectified linear unit) layer, and a sigmoid activation layer, comprised the trainable parameters.

### 2.2. Model Training

#### 2.2.1. Data Collection

The data of “events” were collected: staged incidences or re-enactments of a person entering a room, wearing either all, some, or no PPE. The volunteers ranged from age 12 to 82, from both sexes, varying ethnicities, and different physical dimensions like height and weight. The videos were recorded on a mobile device, either a Pixel 4a (2020 Google, Mountain View, CA, USA) or an iPhone 15 (2023 Apple, Cupertino, CA, USA). Six locations were tested to reduce overfitting and increase robustness.

The field testing of the system was set up in an empty patient room in the Cardiac Intensive Care Unit at Hadassah Ein-Kerem medical center. This served as a qualitative measurement to validate capabilities on unseen data. Thirteen participants included doctors, nurses, and other medical staff. Eleven of them were not present in the training data and were completely new and unseen to the model.

#### 2.2.2. Data Processing and Training

The collected data videos were split into single-frame images, sampling at a ratio of 1:5 to allow variability between the samples. Each image was manually labeled by the current permutation of protective equipment worn in the video. Each image was fed into the pose estimation and cropping process, resulting in 4 images per frame corresponding to: face, torso, right palm, and left palm. Manual data cleaning was performed to remove false images in cases where the wrist was out of the image field and images of the back of the head. We obtained 5674 face images, 10,836 palm images, and 7029 torso images.

Each dataset was split with a validation subset of 20% of its total size. We then used the Keras abstraction interface in TensorFlow to train the custom models for ten epochs, applying the standard “Adam” gradient descent optimizer and the binary cross-entropy loss on the probabilities. The training machine was an M2 MacBook Air with 16 GB RAM, 512 GB SSD, using the metal hardware GPU acceleration for TensorFlow.

### 2.3. System Validation

#### 2.3.1. Inference System

The models were installed on a Raspberry Pi 5 8GB unit connected to a USB webcam (PS3 CamEye) during the live inference testing. Each iteration lasted ~80 ms, matching the live camera feed with the model results

#### 2.3.2. Data Analysis

The percentage of events in which there was agreement was calculated with the Kappa measurement of agreement beyond chance. The McNemar test was applied to test whether there was a difference between the truth and the AI prediction. Finally, to associate between two categorical variables (e.g., sex and agreement), we used the chi-square test.

#### 2.3.3. Statistical Methods

Sample size estimation was performed to build a 95% confidence interval (CI) for the percentage of events in which there was an agreement between the AI prediction and the ground truth. Assuming that, in 80% of the events, there was agreement between those two values (the minimal percent required for a reliable tool [18]) to build a 95% CI that was 10% wide (75–85%), a sample size of at least 246 samples was needed.

## 3. Results

### 3.1. Classification Accuracy

We separately analyzed each of the three AI models and formed “predictions vs. ground truth” confusion matrices (Table 1) based on manually labeling the existence of a PPE item in the cropped image. We then assessed the performance of each of the AI models through their statistical parameters, yielding accuracies of 96.09%, 93.24%, and 97.34%, for the mask, gloves, and gown models respectively. The McNemar scores were equal or above 24.67, and, by calculating the significance as one minus the cumulative distribution function of the score, we obtained *p*-values of less than 0.001.

During live deployment, we yielded accuracies of 89.46%, 89.21%, and 87.85% for the mask, gloves, and gown models. *p*-values remained negligible, under 0.001, for the mask and gown models, yet insignificant for the gloves, with *p* = 0.337.

### 3.2. Assessing Overall Accuracy

We assessed the solution’s global accuracy by considering a result “correct” only if all detected regions—face, palms, and torso—were accurately classified (Table 2). Recognizing that body positioning and movement could obscure regions of interest, we defined a successful image classification as one where all *visible* regions were correctly evaluated for PPE presence. Considering a 95% confidence interval and a 7124 sample size, we obtained an error margin of 0.82 percent for the model’s overall classification capabilities, coined as “no misclassified regions”.

To further refine our analysis, we stratified the results for mask and gown detection. This stratification was crucial because the glove detection model performed the poorest of the three, and we wanted to isolate and evaluate its impact on the overall performance.

### 3.3. Secondary Confounders

In addition to the overall accuracy, we sought to determine the effect of confounding characteristics on the final results (Table 3):

1. Underlying clothing, i.e., scrubs vs. casual clothing, *p*-value = 0.011.
2. Participant’s sex, *p*-value < 0.001 (none identified as nonbinary).
3. Participant’s height, above or below the median height, *p*-value < 0.001.
4. Background scenery, i.e., hospital setting vs. non-hospital setting, *p*-value < 0.001.

## 4. Discussion

We developed a practical, efficient, and cost-effective solution for the real-time monitoring of PPE adherence. We demonstrated that a custom-trained, lightweight AI model could accurately detect the presence of protective items in real-world healthcare settings.

Designing AI models for PPE detection has been extensively studied before, both in the clinical setting [19,20] and in other industries such as construction safety [21,22,23]. This is because the regions of interest remain consistent throughout the different implementations (i.e., face, palms, and torso).

### 4.1. Building the Model

We extracted distinct images from each video, cropped relevant regions, and fed them to their respective classification models. After labeling the data, we trained the models on the entire dataset. The performance of each custom AI model was highly accurate (Table 1), reaching values of 93% and above. This claim is strengthened by the Cohen’s Kappa values, which were all higher than 0.846, suggesting tight agreement between the prediction and the ground truth, hence making it unlikely that this result can be explained by chance alone.

Despite being trained on twice the data, the glove model performed worse than the others. This stemmed from the complexity of isolating the palm compared to the simpler face and torso regions, which were straightforward to obtain from the landmarks. The palm required interpolation from wrist and elbow landmarks, leading to cropped areas often much larger than the actual palm. This introduced extraneous data and noise, diluting the model’s focus on palm-specific features. Furthermore, the variability in wrist and elbow positioning, reflecting the palm’s three-dimensional orientation, added geometric distortion and inconsistency to the dataset, hindering the glove model’s learning.

Regarding the system’s combined accuracy, the baseline was that it could not outperform the least-performing model. The value of 85.58 ± 0.82% is nonetheless better than what we expected, surpassing the 80% clinical threshold we originally considered as sufficient.

### 4.2. Comparison to Existing Solutions

This performance is comparable to that of other real-time solutions [19,22], which achieved an 84.14–86.55% accuracy with a 40 ms processing time. However, our approach uses a more efficient model, optimized for edge computing, in contrast to others that rely on local servers. This distinction highlights a balance between speed and resource utilization: while some models offer faster processing, our model provides a more scalable and cost-effective solution for real-time PPE monitoring.

Recent research has also explored more complex neural network architectures [24,25], achieving high accuracy in complex environments. For instance, the MEAG-YOLO model [25] utilizes a multi-scale feature fusion approach to enhance the detection of PPE in substations, demonstrating the potential for high accuracy in specific industrial settings. Additionally, alternative sensing methods like depth sensors [5] or signal strength indicators [26] have been explored, achieving even higher accuracy levels up to 86.21–100% [5]. However, these methods often require additional hardware, increasing complexity and cost. Our system, while maintaining competitive accuracy, stands out by using a simpler, more streamlined model that can be efficiently deployed on low-cost edge devices. This simplicity not only reduces computational overhead but also makes our solution more practical for widespread adoption in healthcare settings.

### 4.3. Live Deployment and Confounders

While our overall accuracy was promising, we found interesting trends in the data subgroups (Table 3). Clothing type, surprisingly, had a minimal impact. However, sex and height showed expected disparities, due to height differences among the sexes and the 2:1 sample size imbalance. Scenery had a significant impact, likely due to varying lighting and background noise. However, the model’s intended hospital setting should mitigate this issue.

During live deployment, the separate region models maintained favorable accuracy, above 87.85% (Table 2). The global correctness was evidently impacted, as seen in both the lower Kappa scores as well as the severe reduction in the overall product accuracy from 85.58% down to 70.52%. Moreover, the prediction results for the glove model were no longer significant, with *p* = 0.337. When ignoring the glove predictions, aggregating only the mask and gown, we returned to a reassuring 81.81% accuracy. This tightly correlates to the findings in the training phase, signifying the need to better fine-tune the glove detection model, which will ensure acceptable model performance.

Some unexpected and misleading events included instances where the model misidentified doctor’s white coats as single-use surgical gowns, despite not being considered during training. Additionally, while highly contrasted colors like green and white in scrubs proved effective, slightly colored blue tops led to false positives for the gown. Furthermore, the presence of plastic bags around gloved hands or situations where one hand was gloved and the other was not resulted in inaccurate outputs.

Nonetheless, data collection still is a key aspect of model optimization and improvements. The fact that the individual models preserved a high ~88% detection accuracy serves as clear evidence for the possibility that these results can be improved over time, matching and perhaps surpassing the original training values.

### 4.4. Future with Transformer-Based Models

Large AI models, like those used in generative AI tools, serve as off-the-shelf pretrained solutions that perform instant inferences on image or textual inputs. Although rapidly improving, they are complex to fine-tune on a certain task [27] and their immense size requires remote compute resources, posing a threat for data security and confidentiality [28].

Lightweight models offer stronger cyber protection by analyzing the data on the premise, while enabling rapid accuracy improvements through live result feedback loops. Therefore, on-device models are well suited for healthcare infection reduction and may further encourage the development of additional specialized AI-based equipment.

## 5. Conclusions

This study demonstrates the potential of AI-powered computer vision for monitoring PPE adherence in healthcare settings. We developed a novel system for the real-time monitoring of PPE adherence, utilizing AI-powered on-device processing with a single-board computer, achieving an accuracy of 85.58%. The system’s real-time capability was validated with a live deployment that demonstrated 87–89% per-item accuracy. While challenges remain regarding selection bias and accuracy on unseen data, addressing these issues paves the way for a more effective and efficient solution for ensuring proper PPE use and ultimately fostering a safer healthcare environment for both patients and healthcare workers.

Our proposed low-cost system serves a scalable solution that could be spread and installed throughout an entire medical facility. Such a large-scale distribution can strongly benefit the prevention of the spread of disease hospital-wide.

## Figures and Tables

**Table 1:** Per-item true/false confusion matrices of the training and live data AI predictions.

		Mask Model				Glove Model				Gown Model			
Confusion Matrix		Training Prediction		Live Data Prediction		Training Prediction		Live Data Prediction		Training Prediction		Live Data Prediction	
		True	False	True	False	True	False	True	False	True	False	True	False
Ground truth	True	2620	148	476	70	5577	264	1161	168	3725	51	376	29
	False	74	2832	118	1120	468	4527	186	1765	136	3117	239	1561
Accuracy		96.09%		89.46%		93.24%		89.21%		97.34%		87.85%	
Kappa		0.922		0.758		0.864		0.777		0.946		0.663	
McNemar		24.67		12.26		56.85		0.92		38.64		164.55	
*p*-value		<0.001		<0.001		<0.001		0.337		<0.001		<0.001	

**Table 2:** Overall evaluation of the system’s accuracy during training and on live data.

	Training Data		Live Deployment	
Overall	No Misclassified Regions	No Misclassified Mask or Gown	No Misclassified Regions	No Misclassified Mask or Gown
“Correct”	6097	6254	1624	1884
“Incorrect”	1027	870	679	419
Accuracy	85.58%	87.79%	70.52%	81.81%

**Table 3:** Overall system accuracy relative to the secondary confounding subgroups.

	Clothing		Sex		Height		Scenery	
Predict	Scrubs	Casual	Male	Female	>165 cm	<165 cm	Hospital	Regular
Correct	3638	2459	4211	1886	3925	2121	2934	3163
Incorrect	557	470	613	414	550	472	282	745
Accuracy	86.72%	83.95%	87.29%	82%	87.71%	81.8%	91.23%	80.94%
χ²	10.71		35.35		46.51		151.51	
*p*-value	0.0011		<0.001		<0.001		<0.001	

## Data Availability

The original contributions presented in this study are included in this article. Further inquiries can be directed to the corresponding authors.

## Abbreviations

The following abbreviations are used in this manuscript:

PPE	Personal protective equipment
SBC	Single-board computer
AI	Artificial intelligence
